# Molecular Mapping and Genomics of Grain Yield in Durum Wheat: A Review

**DOI:** 10.3390/ijms21197021

**Published:** 2020-09-24

**Authors:** Osvin Arriagada, Ilaria Marcotuli, Agata Gadaleta, Andrés R. Schwember

**Affiliations:** 1 Departamento de Ciencias Vegetales, Facultad de Agronomía e Ingeniería Forestal, Pontificia Universidad Católica de Chile, 306-22 Santiago, Chile; arriagada.lagos.o@gmail.com; 2 Department of Agricultural and Environmental Science, University of Bari Aldo Moro, 70121 Bari, Italy; i.marcotuli@gmail.com (I.M.); agata.gadaleta@uniba.it (A.G.)

**Keywords:** *Triticum turgidum* ssp. *durum*, yield components, QTLs, molecular markers

## Abstract

Durum wheat is the most relevant cereal for the whole of Mediterranean agriculture, due to its intrinsic adaptation to dryland and semi-arid environments and to its strong historical cultivation tradition. It is not only relevant for the primary production sector, but also for the food industry chains associated with it. In Mediterranean environments, wheat is mostly grown under rainfed conditions and the crop is frequently exposed to environmental stresses, with high temperatures and water scarcity especially during the grain filling period. For these reasons, and due to recurrent disease epidemics, Mediterranean wheat productivity often remains under potential levels. Many studies, using both linkage analysis (LA) and a genome-wide association study (GWAS), have identified the genomic regions controlling the grain yield and the associated markers that can be used for marker-assisted selection (MAS) programs. Here, we have summarized all the current studies identifying quantitative trait loci (QTLs) and/or candidate genes involved in the main traits linked to grain yield: kernel weight, number of kernels per spike and number of spikes per unit area.

## 1. Introduction

Durum wheat (*Triticum turgidum* L. ssp. *durum*) is an important crop worldwide with approximately 16 million ha sown annually and a production of ~40 million tons in 2017 [1,2]. However, there is little detail about durum wheat production; in fact, the statistics reported by FAOSTAT do not differentiate among the different wheat species grown in the world. Despite this, it is well known that durum wheat is mainly cultivated in the Mediterranean regions, where the principal producers in the world are Spain, France, Italy and Greece in southern Europe, Morocco, Algeria and Tunisia in northern Africa, Turkey and Syria in southwest Asia and Canada, USA and Mexico in North America [2], and Argentina and Chile have smaller production areas in South America. Although the surface cultivated with durum wheat represents about 10% of the whole area grown with wheat [3], this cereal plays a key role in human nutrition because it is mainly used for the production of pastas, couscous and other semolina-based products, which are widely consumed in many countries of the world [4]. For this reason, new varieties of durum wheat require combining high grain yield and a number of grain quality parameters demanded by pasta producers [5].

Durum wheat provides important cultural and commercial benefits in the Mediterranean regions, but the crop may be seriously affected by climate change, which can produce yield losses of up to 50% [6]. In this context, gaining insight into the genetic basis of economically important traits is essential to increase the genetic gains and to reduce the projected impact of climate change on yields [7]. Recently, Colasuonno et al. [8] and Marcotuli et al. [9] reviewed the genetic improvements of pigment content and fiber, respectively, in durum wheat grain obtained by breeding approaches, which are import quality traits. However, to meet the future demand and consumption of durum wheat, a substantial increase in grain yield is necessary [10]. One of the strategies that allows achieving this objective and having sustainable productivity over time is through the development and release of new varieties with improved yield potential under different environmental conditions [11]. Thus, the development of cultivars with high grain yield has become a major objective of wheat breeding programs worldwide [12,13,14,15,16,17,18]. For purposes of genetic improvement of grain yield, the direct selection of yield is not always is effective. Therefore, the most desirable approach to improve grain yield is through the simultaneous selection based on grain yield-related traits [19]. Different breeding approaches including quantitative trait loci (QTL) and genome-wide association studies (GWAS) are being applied to increase the grain yield potential of durum wheat through molecular marker-assisted selection (MAS). In fact, multiple QTLs with large and small effects have been mapped on all wheat chromosomes and they have been recently published [20,21,22,23,24]. Therefore, due to the importance of this complex trait and to summarize the available information on the genetics of grain yield in durum wheat, the objective of this review is to analyze in a comprehensive manner the most recent findings associated with the genetics, genomics and the identification of QTL for grain yield-related traits in durum wheat.

## 2. Genetics of Grain Yield in Wheat

Unraveling the genetic basis of grain yield can be carried out through the identification of QTLs for yield-related traits under irrigated, hot and dry environments, fine mapping those QTLs and finding molecular markers tightly linked to the QTL/gene for molecular breeding [25]. In this sense, significant progress has been made in the development of DNA-based molecular markers like AFLP, SSR, DArT and SNP, which have been used in the construction of genetic maps and for conducting QTL analysis through different methods such as single-marker analysis, interval mapping and genome-wide association studies, among others. Given that a large number of molecular markers are necessary for higher resolution and precision in the genetic mapping of complex traits, hundreds of thousands of markers have been developed in wheat, including a wheat 90K SNP iSelect assay containing 81,587 SNP markers [26]. SNPs have been widely used to dissect the genetic basis of several economically important traits [15,17,18,20], and to develop a high-density durum wheat consensus map [27].

Most of the QTL mapping studies have been conducted using common wheat (*Triticum aestivum* L.). For example, a QTL meta-analysis performed by Zhang et al. [28] determined that a total of 541 QTLs were detected for yield-related traits across all chromosomes. More recently, Gupta et al. [29] reviewed the available literature on yield genetics and its components and determined that more than 1200 QTLs have been described to date. In this context, one study reported a major stable QTL for grain length on chromosome 4A (QGl-4A), which mapped into a 0.37 cM interval between KASP markers *Xib4A-10* and *Xib4A-12*, corresponding to the 20 Mb physical region in the CS reference genome sequence (IWGSC RefSeq v1.0). This QTL explained up to 17.3% of the phenotypic variation for grain length in two populations of common wheat, suggesting that markers *Xib4A-10* and *Xib4A-12* could be used for marker-assisted selection in breeding [22]. In parallel, recent GWAS studies have been reported attempting to decipher the genetic architecture of wheat grain yield [20,21].

**Figure 1:**
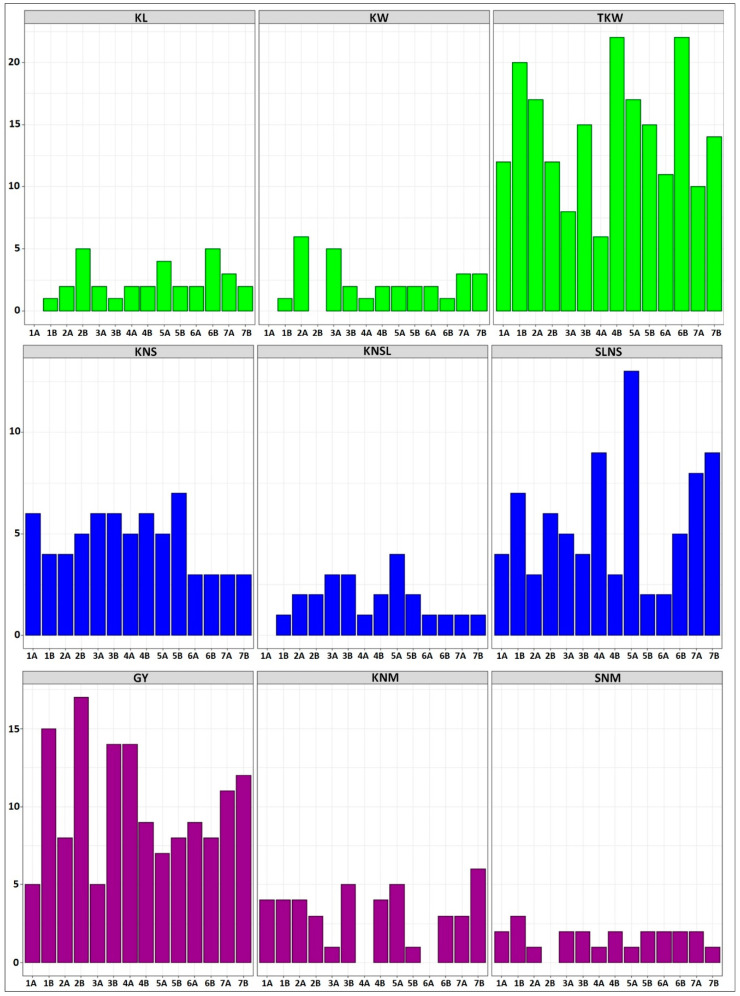
Histograms showing the number of quantitative trait loci (QTLs) detected on each chromosome of durum wheat for each yield-related trait selected and described in this study. Kernel length (KL, 33 QTLs), kernel width (KW, 30 QTLs) and thousand-kernel weight (TKW, 201 QTLs) associated with the kernel weight (in green); kernels number per spike (KNS, 66 QTLs), kernels number per spikelet (KNSL, 24 QTLs) and spikelets number per spike (SLNS, 80 QTLs) associated with kernels number (in blue); grain yield (GY, 142 QTLs), kernels number per square meter (KNM, 43 QTLs) and spike number per square meter (SNM, 23 QTLs) associated with kernels number per unit area (in purple).

In recent decades, the number of studies analyzing the genetic basis of yield in durum wheat has increased. Durum wheat is a tetraploid species (2n = 4x = 28, AABB), comprising an A genome from *T. urartu* and a B genome from *Aegilops speltoides* [30]. In addition, grain yield (GY) is a multifactorial trait determined by multiple genes that interact with each other and with the environment [11]. Thus, the genetic basis of GY has been investigated through the three main yield components which will be explored in this review: kernel weight, kernels number and kernels number per unit area [28,31]. A total of 665 QTLs (100 with a pleiotropic effect) were identified and associated with grain yield and its components (Table S1) [7,14,15,17,18,30,32,33,34,35,36,37,38,39,40,41,42,43,44,45,46,47,48,49,50,51,52,53,54,55,56,57,58,59,60,61,62,63,64]. Specifically, nine additional yield components were considered: kernel length (KL), kernel width (KW), thousand-kernel weight (TKW) associated with the kernel weight, kernels number per spike (KNS), kernels number per spikelet (KNSL), spikelets number per spike (SLNS) associated with the kernels number, grain yield (GY), kernels number per square meter (KNM) and spike number per square meter (SNM) related to the kernels number per unit area (Figure 1). Results from a study revealed that major well-known genes and/or QTLs affecting plant height (PH) and earliness which are related to grain yield traits co-localized with outlier SNP loci in a large panel of Italian durum wheat accessions [24]. Therefore, QTLs for plant height (PH) were also included (Table S1). Using a large Australian wheat population, Joukhadar et al. [23] showed that long-term artificial selection left distinct genomic signatures that can be used to retrospectively understand breeding targets, and to identify economically important alleles, such as the regions including *Glu-B1*, *TaGw2-6A*, *Cre8*, *Ppd-D1*, *Rht-B1*, *Vrn-B1*, *TaSus1-7A*, *TaSAP1-7A* and *Psy-A1* plus multiple QTLs affecting wheat yield and yield components. The recently assembled genome of the Italian durum wheat *cv*. “Svevo” will be very useful for future improvement of the target traits associated with grain yield [65].

### 2.1. Kernel Weight

Among the yield components, TKW is one of the most studied traits because it is less affected by the environment, i.e., more stable heritability, and it is useful for indirect yield improvement [66]. In fact, TKW is the yield-related trait with the highest number of QTLs detected in durum wheat (Figure 1). TKW is determined by grain morphometric parameters (mainly length and width of the grain) and grain-filling rate [67]. In the last ten years, many QTLs for TKW have been identified on all chromosomes based on biparental and association populations in common wheat [29], which has allowed the characterization of genes involved in the kernel length (KL) and kernel width (KW), and the corresponding development of functional markers for MAS. Among them, some of them stand out such as cell wall invertase (*CWI*) genes designated as *TaCWI-A1* [68], *TaCWI-4A* and *TaCWI-5D* [69], which encode a critical enzyme for sink tissue development and carbon partition and have a high association with kernel weight. Grain Weight 2 (*TaGW2*) genes that encode a RING-type protein with E3 ubiquitin ligase activity, which negatively regulates the kernel width and loss-of-function mutations in the coding sequence, result in enhanced kernel width and kernel weight [70,71], among others described below. In this context, the identification of functional markers associated with the genes affecting kernel size and kernel weight in wheat is useful in breeding to increase yields by MAS.

**Figure 2:**
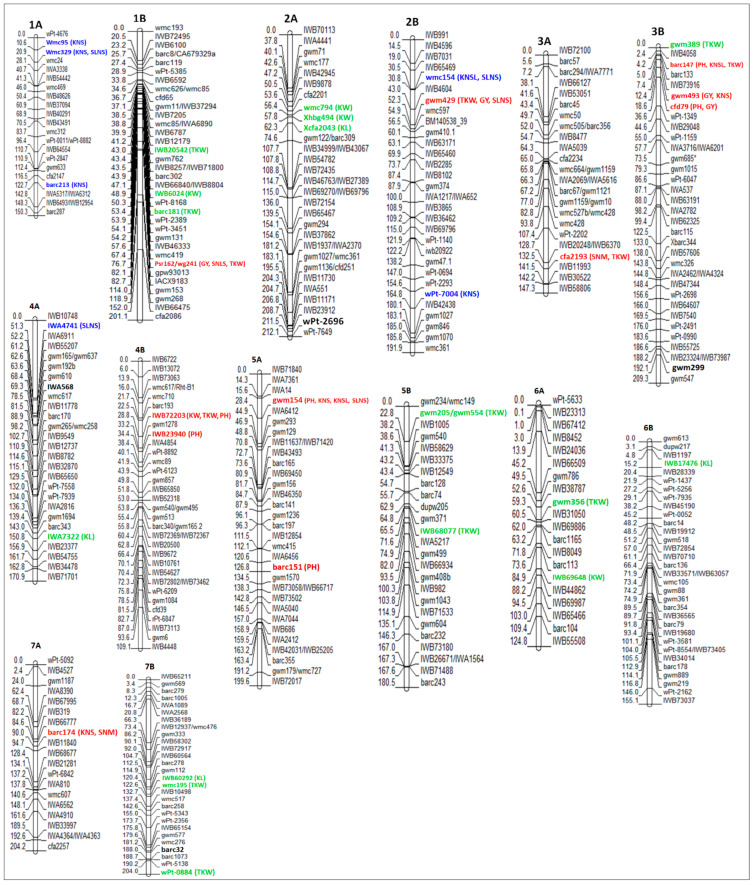
All markers associated with QTLs for grain yield-related traits reported in durum wheat genetic studies, whose marker are present in the consensus map developed by Maccaferri et al. [27]. On the left of the chromosome, the marker position in cM, which corresponds to that of the consensus map, is presented. On the right of the chromosome, the name of the markers and QTLs with a moderate/major effect on kernel weight (in green) and kernels number (in blue) traits is presented. Moreover, major QTLs with a pleiotropic effect on different categories are shown in red. QTLs detected in different populations are shown in bold.

Modern durum wheat varieties exhibit large kernels and normally uniform seed size, because of the domestication and breeding processes that have focused on increasing yields. Conversely, durum wheat landraces show a much greater variability for kernel size and shape ([32], and the reference therein). In durum wheat, there is considerably less information of QTLs controlling kernel weight and most studies have been conducted to decipher the genetic basis of TKW [33,34,35,36,37], and fewer studies have been performed to elucidate the genetic basis of kernel size [17,30,32]. In this review, a total of 264 QTLs for kernel weight-related traits (201 for TKW, 33 for KL and 30 for KW) were identified on all chromosomes of durum wheat, however, no QTLs were reported for KW on chromosomes 1A and 2B, and for KL on chromosome 1A (Table S1, Figure 1). Figure 2 shows all the QTLs found in this literature review, whose associated markers are present in the consensus map developed by Maccaferri et al. [27]. This consensus map was developed using thirteen independent biparental populations of tetraploid wheat, including elite x elite, elite x cultivated emmer and elite x wild emmer, where markers common to two or more populations were included in the final map [27]. In this sense, the QTLs and markers shown in Figure 2 are very likely to segregate in independent durum wheat populations that are used in current breeding programs, including elite populations. Eleven moderate and major QTLs (> 20% of the phenotypic variation) for kernel weight-related traits have been detected on all chromosomes of that consensus map, except on chromosomes 1A, 5A and 7A. However, these major QTLs should be considered with caution in durum wheat breeding programs, since some of them are environment-specific. On the other hand, very few major stable QTLs have been detected in independent populations, for example, at ~50 cM on chromosome 5B, a stable major QTL was detected for TKW in two independent populations whose effects accounted for over 20% of the phenotypic variance [32,38].

Despite the fact that most of the studies to identify QTLs related to grain yield have been carried out in Mediterranean environments, there are QTLs (most of them) that were detected only in a particular environment or cropping season, and others that were identified in more than one environment (stable QTLs). The QTLs detected in more than one environment are of interest to plant breeders, however, they show variation in the magnitude of their effect. For example, the QTL for TKW detected on chromosome 2A was stable in five different environments and explained between 1.4% and 12.9% of the phenotypic variation in each environment [39]. In addition, the moderate QTL located at 43 cM on chromosome 1B and linked to the “IWB20542” marker had an effect of 20.9% of the phenotypic variance for TKW in an RIL population (Zavitan × Svevo) [40]. However, in the same region of chromosome 1B, minor QTLs were identified in independent populations, whose effects were of 2.6% [41] and 3.4–6.2% [39] of the TKW variance. Therefore, some major QTLs reported in the literature should be considered with caution for their potential introgression into durum wheat breeding programs.

Moreover, some QTLs have also a pleiotropic effect. In this review, five major QTLs with pleiotropic effects were found on chromosomes 1B at 76.7 cM, 2B at 52.3 cM, 3A at 132.5 cM, 3B at 4.2 cM and 4B at 28.8 cM (Figure 2), which may be important for improving kernel traits through MAS in durum wheat. Moreover, a total of 11 QTLs distributed on chromosomes 2A, 2B, 4B and 7A were associated significantly with TKW in an RIL population obtained from the cross between “PDW 233” and “Bhalegaon 4”. Among them, four QTLs were consistently reported in three environments: two QTLs linked to the marker *XRht-B1* located on chromosome 4B, explaining ~20% of the variation in TKW, and two QTLs positioned in the intervals *Xgwm71.2*–*Xubc835.4* (on chromosome 2A) and *Xgwm429*–*Xubc812.2* (on chromosome 2B), explaining up to 12.36% and 23.70% of the phenotypic variation in TKW, respectively [35]. Recently, four QTLs were identified on chromosomes 2A, 5A and 7B, explaining between 5.5% and 6.8% of the kernel width variance [30]. The QTLs for KL (at 20.75 cM) and KW (at 66.36 cM) identified on chromosome 7B were localized in the same regions as the *TaCYP78A3* and *TaGW8* genes reported by Ma et al. [72] and Yan et al. [73], respectively, on chromosome 7 of common wheat. Sun et al. [17] performed an association study for kernel-related traits in a worldwide collection of durum wheat germplasm. A total of 54 SNP markers that generated 109 marker–trait associations were identified for 8 kernel traits (including KL, KW and TKW), distributed across almost all chromosomes, except chromosome 1A. Most of the SNPs identified were close to or overlapped with the positions of the kernel weight-related QTLs reported in other studies using different populations of durum wheat, which is in agreement with what is documented in this review where several molecular markers are associated with the same or another trait in different populations (see Table S1). In addition, 54 candidate genes were annotated from the significantly associated markers, which were divided into several categories and most of them encoded metabolism-related enzymes, and some of them are involved in kernel development. Finally, the SNPs *BE500291_5_A_37* (on chromosome 5A) and *BF474023_3_A_Y_425* (on chromosome 3A), whose functional annotations were matched with 1-acyl-sn-glycerol-3-phosphate acyltransferase (*PLS1*) and abscisic acid insensitive like1 protein (*ABIL1*) genes, respectively, might play an important role in kernel development in durum wheat [17]. All QTLs for kernel weight-related traits (KL, KW and TKW), their associated markers and positions on the consensus map of durum wheat are shown in Table S1.

### 2.2. Kernels Number

The number of kernels per spike (KNS) in wheat is variable, around 30 to 70, and it is strongly sensitive to high temperatures and drought [74,75], which reduce the kernels during development and grain filling [76]. Due to the complex nature of kernels number, several QTLs have been identified in wheat, mostly of small effect, associated with kernel number per spikelet (KNSL) [76,77], spikelets per spike (SLNS) [10,78,79,80] and kernels per spike (KNS) [10]. Moreover, many favorable genes from wild relatives of wheat have been transferred into common wheat via distant hybridization to increase the KNS [81]. For example, genes located on chromosome 6P in *Agropyron cristatum* have been introgressed into wheat–*A. cristatum* hybrids, generating a greater number of florets and kernels per spike [82,83,84].

KNS depends on the number of spikelets per spike and the number of florets per spikelet. Therefore, the spike architecture is closely related to wheat kernel production because an increase in the number of kernels on a spike can be achieved by a higher number of kernels per spikelet, but also by a higher number of spikelets [85]. In this sense, breeding strategies may be focused on spike-related traits to improve wheat grain yield potential. In this sense, the genetic gains related to yield in wheat in the last century have mainly been achieved due to a KNS increase rather than a higher kernel size [75,86] since negative correlations have been encountered between kernel weight and kernel number [42]. We found a total of 170 QTLs associated with KNS (66), SLNS (80) and KNSL (24) that have been mapped using biparental and association populations in durum wheat. These QTLs are distributed across all chromosomes, but no QTL for KNSL has been mapped on chromosome 1A to date (Figure 1), in accordance with the study performed by Giunta et al. [16], in which eight QTLs for KNSL were identified on six different chromosomes (2B, 3A, 3B, 4B, 5A and 5B), but none on chromosome 1A. Moreover, the QTL on chromosome 4B explained 12% of the KNSL variance and it was associated with the *Rht-B1* gene.

Eleven genomic regions (eight with a pleiotropic effect) with moderate and major effects for kernels number-related traits were located on chromosomes 1A, 1B, 2B, 3B, 4A and 5A (Figure 2), which can be useful in genetic improvement programs to increase the kernels number in durum wheat. However, it is necessary to consider that the phenotypic correlation between KNS and TKW is negative, whereas the correlations between GY and KNS or TKW are positive. Therefore, an increase in KNS is counterbalanced by the reduction in average kernel weight, and vice versa [43]. For example, a total of 17 QTLs for KNS were co-located with QTLs for TKW, however, this had an opposite effect for each trait [43]. Similarly, a QTL for SLNS on chromosome 2A between the markers *Xgwm71.2* and *Xubc835.4* was concomitantly associated with TKW and test weight (TW). The additive effect of the QTL was positive for TKW and TW, and negative for SLNS [35]. This can be explained considering that the increase in kernel number per unit area or per spike results in a lower availability of photo-assimilates synthesized during grain filling for each kernel, which leads to decreases in individual kernel weight due to competition effects [87]. In addition, four genomic regions on chromosomes 3A (at 43.4 and 93.8 cM), 3B (at 12.4 cM) and 5B (at 103.8 cM) presented co-localization of QTLs for KNS and GY, which explained between 7% and 38% of the phenotypic variance, and the additive effect for both traits was positive [18,44].

### 2.3. Kernels Number per Unit Area

The number of spikes per unit area is another important yield component and it is directly affected by the tiller survival and the tiller number per unit area. Increasing the spike formation rate reduces growth competition and loss of photosynthates from ineffective tillers [88]. The number of spikes is a complex trait affected by genotypic and environmental factors [89]. Planting density is an agronomic practice that affects the spike number per unit area and the yield in wheat. Therefore, an optimal planting density value should be established to maximize wheat yield since a density higher than this optimum leads to competition between plants and a reduction in grain yield. For example, Naseri et al. [90] reported that a planting density of 400 plants/m^2^ had the highest grain yield, number of spikes, number of kernels per spike and harvest index in three durum wheat cultivars than other plant densities such as 300, 350 and 450 plants/ m^2^.

Generally, an increase in the productive tiller number enhances the yield potential over a range of environments [45]. However, the tiller number and the spike number are not always controlled by the same QTLs/genes. Therefore, it is crucial to dissect the genetic basis of the spike number to increase the grain yields [89]. There are limited amounts of studies involving QTL mapping for spike formation rate or spikes number per unit area (SNM) in durum wheat. In addition, this trait reported the lowest number of QTLs (23), and only one QTL located at 90 cM on chromosome 7A, associated with the marker “*Xbarc174*” in the consensus map, had a relevant effect [44] (Figure 2). Soriano et al. [36] identified a stable QTL region for KNM and KW at 42.9–43.9 cM on chromosome 1B. In addition, the marker “*wPt-5092*” located on chromosome 7A was concomitantly associated with KNM and GY, with a positive additive effect for both traits. This result is in accordance with Zaïm et al. [37], who detected four QTLs for SNM on chromosomes 1A, 3B, 5B and 6B, explaining between 6.6% and 13.2% of the phenotypic variance. Moreover, four QTLs for SNM were detected on chromosomes 3A, 4B, 6A and 7A, responsible for 14% and 24% of the SNM variance [44].

Studies conducted across a broad range of water availability and temperature conditions in the Mediterranean regions have evidenced the importance of KNM in determining GY in durum wheat. In this context, KNM is strongly correlated with GY [7,46]. For KNM, Graziani et al. [41] identified a total of 17 QTLs across sixteen Mediterranean environments with contrasting thermo-pluviometric conditions. These QTLs were distributed on 10 out of the 14 chromosomes (except 3A, 4A, 6A and 7A), and they explained individually between 3.3% and 18.8% of the total phenotypic variation. QTLs for KNM co-localized with those for TKW, and generally had an opposite additive effect for each trait. For example, the moderate QTL with a pleiotropic effect for KNM and TKW located on chromosome 4B between the markers *Xgwm6* and *Xcfd54* had an additive effect of 0.36 for TKW and -151.77 for KNM [75]. Further, Sukumaran et al. [7] identified two loci that associated with GY, TKW and KNM of a durum wheat population grown under yield potential. The locus on chromosome 2A (61–70 cM) was associated with TKW and KNM, while the locus on chromosome 2B (78–82 cM) was related to TKW, GY and KNM. In this study, common regions for GY, TKW and KNM were not identified under a drought environment. However, a locus on chromosome 7B (36–40 cM) was associated with GY and KNM. Finally, a locus on chromosome 2B (74–82 cM) was associated with TKW and KNM under heat stress. In summary, these loci and the associated markers can be useful to improve the number of spike/kernel number per unit area in durum wheat grown under different environmental conditions.

Overall, a total of 43 QTLs for KNM have been reported, however, no major QTL has been extrapolated to the consensus map due to the strong correlation that exists between KNM and GY, most of the studies in kernels number per unit area have been carried out for grain yield (GY) and a total of 142 QTLs have been reported in durum wheat, being the second most studied trait after TKW. Moreover, four moderate/major QTLs have been identified in markers present in the consensus map (Figure 2), which can be considered to improve the kernels number in durum wheat through MAS.

Grain yield is the second trait with the most QTLs detected, after TKW. A total of 142 QTLs have been identified for GY across all chromosomes of durum wheat, including five major QTLs with the effect of over 20% of the phenotypic variance (Table S1, Figure 2). Three of them were identified by Roncallo et al. [44] in an RIL population derived from the cross between “UC1113” and “Kofa”. The QTL located in the interval marker “*cfa2201*–*gwm429”* on chromosome 2B at 53.6 cM explained 23% of the GY variance, and the positive allele was contributed by “UC1113”. Additionally, two major QTLs were identified between the intervals “*gwm493–cfd79”* at 12.4 cM and “*cfd79–ksm45”* at 18.6 cM on chromosome 3B, which explained 38% and 23.9% of the GY variation. The positive allele for both QTLs was contributed by “Kofa”, therefore this region on chromosome 3B can be used as a selection criterion in breeding programs of durum wheat that use the “Kofa” cultivar as a parental.

Despite the fact that thousands of QTLs have been identified in wheat for grain yield-related traits, very few QTLs have been used in genetic improvement programs through MAS [29]. Since QTLs for grain yield have been identified on all 14 chromosomes of the durum wheat genome, genomics-based approaches like genomic selection (GS) are suitable to supplement the genetic improvement of this trait [29]. The use of models focused on genomic prediction increases the rates of genetic gain for complex traits, since the genetic values of individuals are estimated with greater precision and reduce the generational intervals between breeding cycles [91]. In wheat, most of the SG studies have been carried out in bread wheat [92,93,94] and very few in durum wheat [5,95,96]. Considering that GS explores the whole genome looking for large and small allelic effects [97], it is a useful approach to complement the genetic improvement of durum wheat grain yield. For example, Haile et al. [98] obtained between 0.5 to 0.8 of accuracy in the prediction of genomic values for traits associated with yield and grain quality in durum wheat. In parallel, Fiedler et al. [99] implemented different SG models, and they reported values between 0.27 and 0.66 of precision for five different traits associated with grain quality. Finally, the information of previously identified QTLs can be included in genomic prediction models to increase the prediction ability [100]. In this sense, Zaïm et al. [37] recently increased the precision in the prediction from 0.37 to 0.54 and 0.30 to 0.54, for grain yield and TKW, respectively, when the QTL-underlying markers were fixed in the model. Therefore, a combined approach between associative mapping and genomic selection allows increasing genetic gains for grain yield-related traits in durum wheat breeding programs.

## 3. Genes Affecting Yield and Its Components in Durum Wheat

The genetic control of grain yield and its components has been well documented in rice, one cereal model species [101]. However, several wheat genes have also been identified using map-based cloning of QTLs and screening of T-DNA tagging libraries [102,103], which regulate cell proliferation and cell elongation and they are involved in several pathways, including the IKU pathway, the ubiquitin–proteasome pathway, G-protein signaling, the mitogen-activated protein kinase signaling pathway, phytohormones and transcriptional regulatory factors [104]. These progresses in rice genomics and isolation of several of the yield-related genes have provided opportunities for homology-based cloning of genes in wheat [105].

The identification of hundreds of QTLs for TKW based on biparental and association populations in common wheat [106,107,108,109,110] has allowed the characterization of genes involved in the kernel-related traits. Among them are cell wall invertase (*CWI*) genes designated as *TaCWI-A1* [68], *TaCWI-4A* and *TaCWI-5D* [69], which encode a critical enzyme for sink tissue development and carbon partition and have a high association with kernel weight. Grain Weight 2 (*TaGW2*) genes that encode a RING-type protein with E3 ubiquitin ligase activity, which negatively regulates the kernel width and loss-of-function mutations in the coding sequence, result in enhanced kernel width and kernel weight [70,71]. In addition, a sucrose synthase type II gene (*TaSus2*) has been significantly associated with TKW [111], and *TaCKX6*, a cytokinin oxidase/dehydrogenase (*CKX2*), has been significantly associated with grain weight [112]. Besides this, a grain size gene (*TaGS5*) that encodes a serine carboxypeptidase positively regulates the grain size by increasing the cell number and, to some extent, the cell size [113,114]. Further, *TaCYP78A3*, a gene encoding the cytochrome P450 CYP78A3 protein, influences the grain size by affecting the extent of integument cell proliferation [72]. In another study, the *TaTGW6* gene was reported to encode a protein with indole-3-acetic acid (IAA)-glucose hydrolase activity, which determines the grain weight [115]. *TaSAP1*, a member of the stress association protein (SAP) gene family in wheat, was significantly associated with grain weight, grain number per spike, spike length and peduncle length in multiple environments [116], among other traits.

Advances have been made in the understanding of the genes regulating kernel size and kernel weight in durum wheat. Moreover, the development of functional markers associated with the genes controlling kernel size and kernel weight is useful in breeding for increasing yields by MAS. For example, Sestili et al. [117] reduced the abundance of the Grain Weight 2 (*GW2*) transcript in the cultivar “Svevo” through the RNAi approach, which resulted in an increase in the kernel width from 4 to 13%. Moreover, Simmonds et al. [118] reported that null mutants for *TaGW2-A1* were associated with an increase in the TKW (6.6%), KW (2.8%) and KL (2.1%) in tetraploid and hexaploid wheat compared to the wild type allele. Additionally, Alemu et al. [30] identified two QTLs for KL (at 20.75 cM) and KW (at 66.36 cM) on chromosome 7B, which were localized in the same regions as the *TaCYP78A3* and *TaGW8* genes reported by Ma et al. [72] and Yan et al. [73], respectively, on chromosome 7 of common wheat. Moreover, Sun et al. [17] annotated a total of 54 candidate genes for kernel-related traits in a worldwide collection of durum wheat germplasm, which were divided into several categories, where most of them encoded metabolism-related enzymes, and some of them were involved in kernel development. They concluded that the SNPs “*BE500291_5_A_37*” (on chromosome 5A) and “*BF474023_3_A_Y_425*” (on chromosome 3A), whose functional annotations were matched with 1-acyl-sn-glycerol-3-phosphate acyltransferase (*PLS1*) and abscisic acid insensitive like1 protein (*ABIL1*) genes, respectively, might play an important role in the kernel development of durum wheat [17].

It is well known that the kernel number and the kernel weight are usually negatively correlated, however, the genetic basis underlying this trade-off has been very little studied. In durum wheat, the locus Grain Number Increase 1 (*GNI1*) is an important contributor to floret fertility. The *GNI-A1* gene encodes a homeodomain leucine zipper class I (HD-Zip I) transcription factor, and mutations or knockdown of this gene lead to an increase in the number of fertile florets and kernel number per spike. Final KW is the result of the interplay between potential kernel weight (sink) and the actual supply of assimilates per kernel during kernel filling (source). Therefore, *GNI-A1* participates in the trade-off between kernel number and kernel weight due to alterations in the assimilate distribution [119,120]. In this sense, Marcotuli et al. [121] identified three putative candidate genes for kernel number per spike. On chromosome 2B, between the SSR markers *Xwmc213* and *Xwmc243*, they identified the APETALA-2-Like transcription factor (*TaAP2*), which plays a central role in the transition phase from vegetative to reproductive growth [122]. On chromosome 3A, the GIGANTEA 3 (*TaGI3*) gene was localized at 0.6 cM from the marker *IWB48828*, which is known to affect the photoperiod pathway and flowering promotion in wheat [123]. The third gene was a 14-3-3 protein (*Ta14A*) that binds to a large number of transcription factors and signaling proteins, participating in the regulation of carbon and nitrogen metabolism, kernel and plant development and biotic and abiotic stress responses [124]. In addition, it has been reported that the ARGONAUTE1d (*AGO1d*) gene controls spike length and kernel number per spike in durum wheat [125].

## 4. Stable QTLs Identified under Stressful Environments

Durum wheat is grown mainly under rainfed conditions in Mediterranean regions. Therefore, it has to cope with water scarcity combined with high temperatures during grain filling, which negatively affects its yield potential [126]. Among the processes affected by high temperatures are the reduced development of the pollen tube, pollen abortion, reduction in the crop cycle, kernel shrinkage, reduction in seed reserves and anther indehiscence, and some physiological processes such as disintegration of chlorophyll and damage to photosystem II of the photosynthetic apparatus, among others [127]. In parallel, the reduction in leaf area, kernel abortion, reduced mobilization of reserves and reduced number of amyloplasts are effects of drought stress in wheat [127]. All these effects on the plant and its development as a result of high temperatures and drought can cause a decrease in the grain yield potential of wheat (review by Fahad et al. [128]).

In Mediterranean regions, rainfall can likely decrease by up to 24% and the temperatures can increase by 1 to 3 °C during the cropping season [129]. Therefore, the release of new cultivars that are more productive and tolerant to these adverse climatic conditions for grain yield is required. Dettori et al. [6] simulated climate change impacts on the production and phenology of durum wheat grown in Mediterranean environments. An increase in temperature of 6 °C and a reduction in annual rainfall of 30% cause a reduction in grain yield between 4 and 32%. In this sense, gaining insight into the genetic basis of the responses to drought and heat stress is an important prerequisite for improvement of durum wheat genotypes, and plant breeders should look for stable loci to improve yields.

The genetics of tolerance under drought and high-temperature conditions has been extensively studied in wheat, however most of the QTLs have been identified in common wheat [29,130]. In durum wheat, an RIL population derived from the cross between Langdon and wild emmer (acc. #G18-16) was used to detect QTLs of this population grown under well-watered and water-limited conditions, and a total of 34 QTLs related to productivity were reported [47]. Among them, six significant QTLs for GY were identified, two were environment-specific and four were stable in both environments. Since durum wheat is mainly grown in Mediterranean environments, the drought and heat stresses are the main abiotic factors that limit the growth and final yield in durum wheat [98]. Moreover, given that GY is a complex trait considerably affected by the genotype x environment interaction, the stable QTLs also show variations in their effects across the environments in which they have been detected. For example, in the study performed by Peleg et al. [47], four stable QTLs were detected under well-watered and water-limited conditions on chromosomes 2B (2), 4A and 4B, which explained between 4.2% and 15.5% of the phenotypic variance. On the contrary, the QTL on chromosome 2B close to the markers *Xgwm374* (at ~69.4 cM) and *wPt-0694* (at ~129.6 cM) had a stable effect of 13.1 and 15.5%, respectively, on the phenotypic variance in both environments. These QTLs may be of particular interest to durum wheat breeders, who are interested in identifying stable QTLs with a similar effect in different environments of the Mediterranean regions.

Sukumaran et al. [7] performed the first and unique comprehensive study on marker–trait associations for grain yield and its components under yield potential, drought and heat conditions in durum wheat. A panel of 208 lines composed of elite materials and exotics from the International Maize and Wheat Improvement Center (CIMMYT) was used. Interestingly, several genomic regions were identified under two environments and in a combined analysis of three environments. Among them, a locus located on chromosome 2A at 66–70 cM was associated with TKW and KNM, and another locus on chromosome 7A at 75 cM was associated with GY and KNM under yield potential and heat conditions. Under drought and heat conditions, a total of 93 QTLs were reported, however, no common QTL for GY, TKW and KNM was identified. In a combined analysis of the three conditions, a total of 124 QTLs were detected. The locus located on chromosome 2A (66–70 cM) was associated with TKW and KNM. Moreover, two loci on chromosomes 2B (82 cM) and 5B (48 cM) were associated with TKW, but not with GY. Similarly, loci on four chromosomes (2A, 5B, 7A and 7B) were identified for KNM but not for GY. These stable loci across environments can be useful in the gene discovery and marker-assisted selection for increasing grain yields in durum wheat in the productive areas affected by climate change. More recently, two genomics regions located on chromosomes 2A and 7B linked with the markers *Xgwm895* (at 125 cM) and *Xbarc276* (at 219 cM) were associated with GY in an RIL population derived from the cross between “Omrabi” and “Belikh2”. These markers can increase the GY up to 6.16% and 5.27% under irrigated and drought conditions, and in a combined manner, a yield rise of up to 11% is possible [18]. However, it is necessary to verify if these markers segregate in elite populations used in genetic improvement programs of durum wheat. An interesting QTL associated with TKW, GY and PH was detected on chromosome 3B between the markers *Xbarc133* and *Xgwm493* in an RIL population derived from the cross between elite cultivars Kofa and Svevo. This QTL was stable under irrigated and rainfed conditions and explained up to 18% of the phenotypic variance for GY [14,41].

Finally, the stable QTLs identified to date for grain yield-related traits of durum wheat grown under normal or irrigated, drought and high-temperature conditions are shown in Table S2 [7,14,18,33,35,47,79,131]. Therefore, these genomic regions are important to study (fine mapping and characterization of genes) for durum wheat breeding programs to produce new varieties well-adapted to drought stress and/or high temperatures, a relevant productive problem in most of the regions where durum wheat is cultivated.

## 5. Future Perspectives and Conclusions

The identification of genes/regions controlling the grain yield in durum wheat requires many efforts due to polyploidy of the species and the quantitative nature of the trait. The information summarized in this review highlights the results obtained by the scientific community on the genetic regions that regulate grain yield. Clearly, the most studied traits affecting grain yield have been the kernel weight, number of kernels per spike and number of spikes per unit area. Many genes have been identified as associated with the detected QTLs and are mainly involved in the regulation of cell proliferation and cell elongation, flowering control, spike elongations and genes for transcription factors and signaling proteins, participating in the regulation of carbon and nitrogen metabolism, kernel and plant development and biotic and abiotic stress responses. Due to the actual growth condition of durum wheat production and the connection between genes for stress responses associated with grain yield QTLs, here we reported all the stable loci under high-temperature and drought conditions. The identification of these stable QTLs allows a better understanding of the regions/genes regulating yield in durum and the development of functional markers, which can be used in breeding programs for yield increases through MAS.

## Supplementary Materials

Supplementary materials can be found at https://www.mdpi.com/1422-0067/21/19/7021/s1.

## References

[1] Dahl C. Global Durum Outlook. http://www.italmopa.com/wp-content/uploads/2017/05/144_all_1.pdf.

[2] Martínez-Moreno F., Solís I., Noguero D., Blanco A., Özberk İ., Nsarellah N., Elias E., Mylonas I., Soriano J.M. (2020). Durum wheat in the Mediterranean Rim: Historical evolution and genetic resources. Genet. Resour. Crop Evol..

[3] Kabbaj H., Sall A.T., Al-Abdallat A., Geleta M., Amri A., Filali-Maltouf A., Belkadi B., Ortiz R., Bassi F.M. (2017). Genetic diversity within a global panel of durum wheat (*Triticum durum*) landraces and modern germplasm reveals the history of allele’s exchange. Front. Plant Sci..

[4] Sharma J.S., Running K.L.D., Xu S.S., Zhang Q., Peters Haugrud A.R., Sharma S., McClean P.E., Faris J.D. (2019). Genetic analysis of threshability and other spike traits in the evolution of cultivated emmer to fully domesticated durum wheat. Mol. Genet. Genom..

[5] Rapp M., Lein V., Lacoudre F., Lafferty J., Müller E., Vida G., Bozhanova V., Ibraliu A., Thorwarth P., Piepho H.P. (2018). Simultaneous improvement of grain yield and protein content in durum wheat by different phenotypic indices and genomic selection. Theor. Appl. Genet..

[6] Dettori M., Cesaraccio C., Duce P. (2017). Simulation of climate change impacts on production and phenology of durum wheat in Mediterranean environments using CERES-Wheat model. Field Crop. Res..

[7] Sukumaran S., Reynolds M.P., Sansaloni C. (2018). Genome-wide association analyses identify QTL hotspots for yield and component traits in durum wheat grown under yield potential, drought, and heat stress environments. Front. Plant Sci..

[8] Colasuonno P., Marcotuli I., Blanco A., Maccaferri M., Condorelli G.E., Tuberosa R., Parada R., de Camargo A.C., Schwember A.R., Gadaleta A. (2019). Carotenoid Pigment Content in Durum Wheat (*Triticum turgidum* L. var *durum*): An Overview of Quantitative Trait Loci and Candidate Genes. Front. Plant Sci..

[9] Marcotuli I., Colasuonno P., Hsieh Y.S.Y., Fincher G.B., Gadaleta A. (2020). Non-Starch Polysaccharides in Durum Wheat: A Review. Int. J. Mol. Sci..

[10] Würschum T., Leiser W.L., Langer S.M., Tucker M.R., Longin C.F.H. (2018). Phenotypic and genetic analysis of spike and kernel characteristics in wheat reveals long-term genetic trends of grain yield components. Appl. Genet..

[11] Sakuma S., Schnurbusch T. (2020). Of floral fortune: Tinkering with the grain yield potential of cereal crops. New Phytol..

[12] Del Moral L.G., Rharrabti Y., Villegas D., Royo C. (2003). Evaluation of grain yield and its components in durum wheat under Mediterranean conditions: An ontogenic approach. Agron. J..

[13] Foulkes M.J., Snape J.W., Shearman V.J., Reynolds M.P., Gaju O., Sylvester-Bradley R. (2007). Genetic progress in yield potential in wheat: Recent advances and future prospects. J. Agric. Sci. Camb..

[14] Maccaferri M., Sanguineti M.C., Corneti S., Ortega J.L.A., Salem M.B., Bort J., DeAmbrogio E., del Moral L.F.G., Demontis A., El-Ahmed A. (2008). Quantitative trait loci for grain yield and adaptation of durum wheat (*Triticum durum* Desf.) across a wide range of water availability. Genetics.

[15] N’Diaye A., Haile J.K., Nilsen K.T., Walkowiak S., Ruan Y., Singh A.K., Clarke F.R., Clarke J.M., Pozniak C.J. (2018). Haplotype Loci Under Selection in Canadian Durum Wheat Germplasm Over 60 Years of Breeding: Association With Grain Yield, Quality Traits, Protein Loss, and Plant Height. Front. Plant Sci..

[16] Giunta F., Pruneddu G., Motzo R. (2019). Grain yield and grain protein of old and modern durum wheat cultivars grown under different cropping systems. Field Crop. Res..

[17] Sun L., Huang S., Sun G., Zhang Y., Hu X., Nevo E., Peng J., Sun D. (2020). SNP-based association study of kernel architecture in a worldwide collection of durum wheat germplasm. PLoS ONE.

[18] Rehman Arif M.A., Attaria F., Shokat S., Akram S., Waheed M.Q., Arif A., Börner A. (2020). Mapping of QTLs Associated with Yield and Yield Related Traits in Durum Wheat (*Triticum durum* Desf.) Under Irrigated and Drought Conditions. Int. J. Mol. Sci..

[19] Ramazani S.H.R., Abdipour M. (2019). Statistical Analysis of Grain Yield in Iranian Cultivars of Barley (*Hordeum vulgare*). Agric. Res..

[20] Li F., Wen W., He Z., Liu J., Jin H., Cao S., Geng H., Yan J., Zhang P., Wan Y. (2018). Genome-wide linkage mapping of yield-related traits in three Chinese bread wheat populations using high-density SNP markers. Theor. Appl. Genet..

[21] Li F., Wen W., Liu J., Zhang Y., Cao S., He Z., Rasheed A., Jin H., Zhang C., Yan J. (2019). Genetic architecture of grain yield in bread wheat based on genome-wide association studies. BMC Plant Biol..

[22] Cao P., Liang X., Zhao H., Feng B., Xu E., Wang L., Hu Y. (2019). Identification of the quantitative trait loci controlling spike-related traits in hexaploid wheat (*Triticum aestivum* L.). Planta.

[23] Joukhadar R., Gendall A.R., Matthew J.H. (2019). Artificial selection causes significant linkage disequilibrium among multiple unlinked genes in Australian wheat. Evol. Appl..

[24] Taranto F., D’Agostino N., Rodriguez M., Pavan S., Minervini A.P., Pecchioni N., Papa R., De Vita P. (2020). Whole Genome Scan Reveals Molecular Signatures of Divergence and Selection Related to Important Traits in Durum Wheat Germplasm. Front. Genet..

[25] Tura H., Edwards J., Gahlaut V., Garcia M., Sznajder B., Baumann U., Shahinnia F., Reynolds M., Langridge P., Balyan H.S. (2020). QTL analysis and fine mapping of a QTL for yield-related traits in wheat grown in dry and hot environments. Theor. Appl. Genet..

[26] Wang S., Wong D., Forrest K., Allen A., Chao S., Huang B.E., Maccaferri M., Salvi S., Milner S.G., Cattivelli L. (2014). Characterization of polyploid wheat genomic diversity using a high-density 90 000 single nucleotide polymorphism array. Plant Biotechnol. J..

[27] Maccaferri M., Ricci A., Salvi S., Milner S.G., Noli E., Martelli P.L., Casadio R., Akhunov E., Scalabrin S., Vendramin V. (2015). A high-density, SNP-based consensus map of tetraploid wheat as a bridge to integrate durum and bread wheat genomics and breeding. Plant Biotechnol. J..

[28] Zhang L., Liu D.C., Guo X.L., Yang W.L., Sun J.Z., Wang D.W., Zhang A. (2010). Genomic distribution of quantitative trait loci for yield and yield-related traits in common wheat. J. Integr. Plant Biol..

[29] Gupta P.K., Balyan H.S., Sharma S., Kumar R. (2020). Genetics of yield, abiotic stress tolerance and biofortification in wheat (*Triticum aestivum* L.). Theor. Appl. Genet..

[30] Alemu A., Feyissa T., Tuberosa R., Maccaferri M., Sciara G., Letta T., Abeyo B. (2020). Genome-wide association mapping for grain shape and color traits in Ethiopian durum wheat (*Triticum turgidum* ssp. *durum*). Crop J..

[31] Yang L., Zhao D., Meng Z., Xu K., Yan J., Xia X., Cao S., Tian Y., He Z., Zhang Y. (2020). QTL mapping for grain yield-related traits in bread wheat via SNP-based selective genotyping. Theor. Appl. Genet..

[32] Desiderio F., Zarei L., Licciardello S., Cheghamirza K., Farshadfar E., Virzi N., Sciacca F., Bagnaresi P., Battaglia R., Guerra D. (2019). Genomic regions from an Iranian landrace increase kernel size in durum wheat. Front. Plant Sci..

[33] Golabadi M., Arzani A., Maibody S.M., Tabatabaei B.S., Mohammadi S.A. (2011). Identification of microsatellite markers linked with yield components under drought stress at terminal growth stages in durum wheat. Euphytica.

[34] Blanco A., Mangini G., Giancaspro A., Giove S., Colasuonno P., Simeone R., Signorile A., De Vita P., Mastrangelo A.M., Cattivelli L. (2012). Relationships between grain protein content and grain yield components through quantitative trait locus analyses in a recombinant inbred line population derived from two elite durum wheat cultivars. Mol. Breed..

[35] Patil R.M., Tamhankar S.A., Oak M.D., Raut A.L., Honrao B.K., Rao V.S., Misra S.C. (2013). Mapping of QTL for agronomic traits and kernel characters in durum wheat (*Triticum durum* Desf.). Euphytica.

[36] Soriano J.M., Malosetti M., Roselló M., Sorrells M.E., Royo C. (2017). Dissecting the old Mediterranean durum wheat genetic architecture for phenology, biomass and yield formation by association mapping and QTL meta-analysis. PLoS ONE.

[37] Zaïm M., Kabbaj H., Kehel Z., Gorjanc G., Filali-Maltouf A., Belkadi B., Nachit M.M., Bassi F.M. (2020). Combining QTL analysis and genomic predictions for four durum wheat populations under drought conditions. Front. Genet..

[38] Peng J.H., Ronin Y., Fahima T., Röder M.S., Li Y., Nevo E., Korol A. (2003). Domestication quantitative trait loci in *Triticum dicoccoides*, the progenitor of wheat. Proc. Natl. Acad. Sci. USA.

[39] Fatiukha A., Filler N., Lupo I., Lidzbarsky G., Klymiuk V., Korol A.B., Pozniak C., Fahima T., Krugman T. (2020). Grain protein content and thousand kernel weight QTLs identified in a durum× wild emmer wheat mapping population tested in five environments. Theor. Appl. Genet..

[40] Avni R., Oren l., Shabtay G., Assili S., Pozniak C., Hale I., Ben-Davis R., Peleg Z., Distelfeld A. (2018). Genome based meta-QTL analysis of grain weight in tetraploid wheat identifies rare alleles of *GRF4* associated with larger grains. Genes.

[41] Graziani M., Maccaferri M., Royo C., Salvatorelli F., Tuberosa R. (2014). QTL dissection of yield components and morpho-physiological traits in a durum wheat elite population tested in contrasting thermo-pluviometric conditions. Crop Pasture Sci..

[42] Gegas V.C., Nazari A., Griffiths S., Simmonds J., Fish L., Orford S., Sayers L., Doonan J.H., Snape J.W. (2010). A genetic framework for grain size and shape variation in wheat. Plant Cell.

[43] Mangini G., Gadaleta A., Colasuonno P., Marcotuli I., Signorile A.M., Simeone R., De Vita P., Mastrangelo A.M., Laidò G., Pecchioni N. (2018). Genetic dissection of the relationships between grain yield components by genome-wide association mapping in a collection of tetraploid wheats. PLoS ONE.

[44] Roncallo P.F., Akkiraju P.C., Cervigni G.L., Echenique V.C. (2017). QTL mapping and analysis of epistatic interactions for grain yield and yield-related traits in *Triticum turgidum* L. var. *durum*. Euphytica.

[45] Naruoka Y., Talbert L.E., Lanning S.P., Blake N.K., Martin J.M., Sherman J.D. (2011). Identification of quantitative trait loci for productive tiller number and its relationship to agronomic traits in spring wheat. Theor. Appl. Genet..

[46] Maccaferri M., Sanguineti M.C., Demontis A., El-Ahmed A., Del Moral L.G., Maalouf F., Nachit M., Nserallah N., Ouabbou H., Rhouma S. (2011). Association mapping in durum wheat grown across a broad range of water regimes. J. Exp. Bot..

[47] Peleg Z., Cakmak I., Ozturk L., Yazici A., Jun Y., Budak H., Korol A.B., Fahima T., Saranga Y. (2009). Quantitative trait loci conferring grain mineral nutrient concentrations in durum wheat× wild emmer wheat RIL population. Theor. Appl. Genet..

[48] Dura S.A., Duwayri M.A., Nachit M.M. (2013). Detection of molecular markers associated with yield and yield components in durum wheat (*Triticum turgidum* L. var. *durum* Desf.) under drought conditions. Afr. J. Agric. Res..

[49] Faris J.D., Zhang Q., Chao S., Zhang Z., Xu S.S. (2014). Analysis of agronomic and domestication traits in a durum× cultivated emmer wheat population using a high-density single nucleotide polymorphism-based linkage map. Theor. Appl. Genet..

[50] Kidane Y.G., Hailemariam B.N., Mengistu D.K., Fadda C., Pè M.E., Dell’Acqua M. (2017). Genome-wide association study of *Septoria tritici* blotch resistance in Ethiopian durum wheat landraces. Front. Plant Sci..

[51] Elouafi I., Nachit M.M. (2004). A genetic linkage map of the Durum× *Triticum dicoccoides* backcross population based on SSRs and AFLP markers, and QTL analysis for milling traits. Theor. Appl. Genet..

[52] Giancaspro A., Giove S.L., Zito D., Blanco A., Gadaleta A. (2016). Mapping QTLs for Fusarium head blight resistance in an interspecific wheat population. Front. Plant Sci..

[53] Giraldo P., Royo C., González M., Carrillo J.M., Ruiz M. (2016). Genetic diversity and association mapping for agromorphological and grain quality traits of a structured collection of durum wheat landraces including subsp. *durum*, *turgidum* and *diccocon*. PLoS ONE.

[54] Giunta F., De Vita P., Mastrangelo A.M., Sanna G., Motzo R. (2018). Environmental and genetic variation for yield-related traits of durum wheat as affected by development. Front. Plant Sci..

[55] Golan G., Oksenberg A., Peleg Z. (2015). Genetic evidence for differential selection of grain and embryo weight during wheat evolution under domestication. J. Exp. Bot..

[56] Iannucci A., Marone D., Russo M.A., De Vita P., Miullo V., Ferragonio P., Blanco A., Gadaleta A., Mastrangelo A.M. (2017). Mapping QTL for root and shoot morphological traits in a Durum Wheat× *T. dicoccum* segregating population at seedling stage. Int. J. Genomics.

[57] Maccaferri M., El-Feki W., Nazemi G., Salvi S., Canè M.A., Colalongo M.C., Stefanelli S., Tuberosa R. (2016). Prioritizing quantitative trait loci for root system architecture in tetraploid wheat. J. Exp. Bot..

[58] Mengistu D.K., Kidane Y.G., Catellani M., Frascaroli E., Fadda C., Pè M.E., Dell’Acqua M. (2016). High-density molecular characterization and association mapping in Ethiopian durum wheat landraces reveals high diversity and potential for wheat breeding. Plant Biotechnol. J..

[59] Milner S.G., Maccaferri M., Huang B.E., Mantovani P., Massi A., Frascaroli E., Tuberosa R., Salvi S. (2016). A multiparental cross population for mapping QTL for agronomic traits in durum wheat (*T riticum turgidum* ssp. *durum*). Plant Biotechnol. J..

[60] Peleg Z., Fahima T., Korol A.B., Abbo S., Saranga Y. (2011). Genetic analysis of wheat domestication and evolution under domestication. J. Exp. Bot..

[61] Russo M.A., Ficco D.B.M., Laido G., Marone D., Papa R., Blanco A., Gadaleta A., De Vita P., Mastrangelo A.M. (2014). A dense durum wheat× *T. dicoccum* linkage map based on SNP markers for the study of seed morphology. Mol. Breed..

[62] Tzarfati R., Barak V., Krugman T., Fahima T., Abbo S., Saranga Y., Korol A.B. (2014). Novel quantitative trait loci underlying major domestication traits in tetraploid wheat. Mol. Breed..

[63] Wang S., Xu S.S., Chao S., Sun Q., Liu S., Xia G. (2019). A genome-wide association study of highly heritable agronomic traits in durum wheat. Front. Plant Sci..

[64] Hu X., Ren J., Ren X., Huang S., Sabiel S.A., Luo M., Nevo E., Fu C., Peng J., Sun D. (2015). Association of agronomic traits with SNP markers in durum wheat (*Triticum turgidum* L. *durum* (Desf.)). PLoS ONE.

[65] Maccaferri M., Harris N.S., Twardziok S.O., Pasam R.K., Gundlach H., Spannagl M., Ormanbekova D., Lux T., Prade V.M., Milner S.G. (2019). Durum wheat genome highlights past domestication signatures and future improvement targets. Nat. Genet..

[66] Xu Y.F., Li S.S., Li L.H., Ma F.F., Fu X.Y., Shi Z.L., Xu H.X., Ma P.T., An D.G. (2017). QTL mapping for yield and photosynthetic related traits under different water regimes in wheat. Mol. Breed..

[67] Simmonds J., Scott P., Leverington-Waite M., Turner A.S., Brinton J., Korzun V., Snape J., Uauy C. (2014). Identification and independent validation of a stable yield and thousand grain weight QTL on chromosome 6A of hexaploid wheat (*Triticum aestivum* L.). BMC Plant Biol..

[68] Ma D., Yan J., He Z., Wu L., Xia X. (2012). Characterization of a cell wall invertase gene *TaCwi-A1* on common wheat chromosome 2A and development of functional markers. Mol. Breed..

[69] Jiang Y., Jiang Q., Hao C., Hou J., Wang L., Zhang H., Zhang S., Chen X., Zhang X. (2015). A yield-associated gene *TaCWI*, in wheat: Its function, selection and evolution in global breeding revealed by haplotype analysis. Theor. Appl. Genet..

[70] Su Z., Hao C., Wang L., Dong Y., Zhang X. (2011). Identification and development of a functional marker of *TaGW2* associated with grain weight in bread wheat (*Triticum aestivum* L.). Theor. Appl. Genet..

[71] Qin L., Hao C., Hou J., Wang Y., Li T., Wang L., Ma Z., Zhang X. (2014). Homologous haplotypes, expression, genetic effects and geographic distribution of the wheat yield gene *TaGW2*. BMC Plant Biol..

[72] Ma M., Wang Q., Li Z., Cheng H., Li Z., Liu X., Song W., Appels R., Zhao H. (2015). Expression of *TaCYP78A3*, a gene encoding cytochrome P450 CYP 78A3 protein in wheat (*Triticum aestivum* L.), affects seed size. Plant J..

[73] Yan X., Zhao L., Ren Y., Dong Z., Cui D., Chen F. (2019). Genome-wide association study revealed that the *TaGW8* gene was associated with kernel size in Chinese bread wheat. Sci. Rep..

[74] Wu X., Chang X., Jing R. (2012). Genetic insight into yield-associated traits of wheat grown in multiple rain-fed environments. PLoS ONE.

[75] Philipp N., Weichert H., Bohra U., Weschke W., Schulthess A.W., Weber H. (2018). Grain number and grain yield distribution along the spike remain stable despite breeding for high yield in winter wheat. PLoS ONE.

[76] Mohammadi M., Karimizadeh R., Shefazadeh M.K., Sadeghzadeh B. (2011). Statistical analysis of durum wheat yield under semi-warm dry land condition. Aust. J. Crop Sci..

[77] Wang J., Liao X., Li Y., Zhou R., Yang X., Gao L., Jia J. (2010). Fine mapping a domestication-related QTL for spike-related traits in a synthetic wheat. Genome.

[78] Zhou Y., Conway B., Miller D., Marshall D., Cooper A., Murphy P., Chao S., Brown-Guedira G., Costa J. (2017). Quantitative trait loci mapping for spike characteristics in hexaploid wheat. Plant Genome.

[79] Cui F., Ding A., Li J., Zhao C., Wang L., Wang X., Qi X., Li X., Li G., Gao J. (2012). QTL detection of seven spike-related traits and their genetic correlations in wheat using two related RIL populations. Euphytica.

[80] Kuzay S., Xu Y., Zhang J., Katz A., Pearce S., Su Z., Fraser M., Anderson J.A., Brown-Guedira G., DeWitt N. (2019). Identification of a candidate gene for a QTL for spikelet number per spike on wheat chromosome arm 7AL by high-resolution genetic mapping. Theor. Appl. Genet..

[81] Zhang Z., Han H., Liu W., Song L., Zhang J., Zhou S., Yang X., Li X., Li L. (2019). Deletion mapping and verification of an enhanced-grain number per spike locus from the 6PL chromosome arm of *Agropyron cristatum* in common wheat. Theor. Appl. Genet..

[82] Wu J., Yang X., Wang H., Li H., Li L., Li X., Liu W. (2006). The introgression of chromosome 6P specifying for increased numbers of florets and kernels from *Agropyron cristatum* into wheat. Theor. Appl. Genet..

[83] Zhang J., Zhang J.P., Liu W.H., Wu X.Y., Yang X.M., Li X.Q., Lu Y.Q., Li L.H. (2016). An intercalary translocation from *Agropyron cristatum* 6P chromosome into common wheat confers enhanced kernel number per spike. Planta.

[84] Zhang J., Ma H.H., Zhang J.P., Zhou S.H., Han H.M., Liu W.H., Li X.Q., Yang X.M., Li L.H. (2018). Molecular cytogenetic characterization of an *Agropyron cristatum* 6PL chromosome segment conferring superior kernel traits in wheat. Euphytica.

[85] Yao H., Xie Q., Xue S., Luo J., Lu J., Kong Z., Wang Y., Zhai W., Lu N., Wei R. (2019). HL2 on chromosome 7D of wheat (*Triticum aestivum* L.) regulates both head length and spikelet number. Theor. Appl. Genet..

[86] Álvaro F., Isidro J., Villegas D., Del Moral L.F.G., Royo C. (2008). Old and modern durum wheat varieties from Italy and Spain differ in main spike components. Field Crop Res..

[87] Foulkes M.J., Slafer G.A., Davies W.J., Berry P.M., Sylvester-Bradley R., Martre P., Calderini D.F., Griffiths S., Reynolds M.P. (2011). Raising yield potential of wheat. III. Optimizing partitioning to grain while maintaining lodging resistance. J. Exp. Bot..

[88] Hu Y.S., Ren T.H., Li Z., Tang Y.Z., Ren Z.L., Yan B.J. (2017). Molecular mapping and genetic analysis of a QTL controlling spike formation rate and tiller number in wheat. Gene.

[89] Li J., Wen S., Fan C., Zhang M., Tian S., Kang W., Zhao W., Bi C., Wang Q., Lu S. (2020). Characterization of a major quantitative trait locus on the short arm of chromosome 4B for spike number per unit area in common wheat (*Triticum aestivum* L.). Theor. Appl. Genet..

[90] Naseri R., Soleymanifard A., Khoshkhabar H., Mirzaei A., Nazaralizadeh K. (2012). Effect of plant density on grain yield, yield components and associated traits of three durum wheat cultivars in Western Iran. Int. J. Agric. Crop Sci..

[91] Daetwyler H.D., Calus M.P., Pong-Wong R., de los Campos G., Hickey J.M. (2013). Genomic prediction in animals and plants: Simulation of data, validation, reporting, and benchmarking. Genetics.

[92] Crossa J., de Los Campos G., Pérez P., Gianola D., Burgueño J., Araus J.L., Makumbi D., Singh R.P., Dreisigacker S., Yan J. (2010). Prediction of genetic values of quantitative traits in plant breeding using pedigree and molecular markers. Genetics.

[93] González-Camacho J.M., de Los Campos G., Pérez P., Gianola D., Cairns J.E., Mahuku G., Crossa J. (2012). Genome-enabled prediction of genetic values using radial basis function neural networks. Theor. Appl. Genet..

[94] Juliana P., Montesinos-López O.A., Crossa J., Mondal S., Pérez L.G., Poland J., Huerta-Espino J., Crespo-Herrera L., Govindan V., Dreisigacker S. (2019). Integrating genomic-enabled prediction and high-throughput phenotyping in breeding for climate-resilient bread wheat. Theor. Appl. Genet..

[95] Crossa J., de los Campos G., Maccaferri M., Tuberosa R., Burgueño J., Pérez-Rodríguez P. (2016). Extending the marker× environment interaction model for genomic-enabled prediction and genome-wide association analysis in durum wheat. Crop Sci..

[96] Mérida-García R., Liu G., He S., Gonzalez-Dugo V., Dorado G., Gálvez S., Solís I., Zarco-Tejada P.J., Reif J.C., Hernandez P. (2019). Genetic dissection of agronomic and quality traits based on association mapping and genomic selection approaches in durum wheat grown in Southern Spain. PLoS ONE.

[97] Bassi F.M., Bentley A.R., Charmet G., Ortiz R., Crossa J. (2016). Breeding schemes for the implementation of genomic selection in wheat (*Triticum* spp.). Plant Sci..

[98] Haile J.K., N’Diaye A., Clarke F., Clarke J., Knox R., Rutkoski J., Bassi F.M., Pozniak C.J. (2018). Genomic selection for grain yield and quality traits in durum wheat. Mol. Breed..

[99] Fiedler J.D., Salsman E., Liu Y., Michalak de Jiménez M., Hegstad J.B., Chen B., Manthey F.A., Chao S., Xu S., Elias E.M. (2017). Genome-wide association and prediction of grain and semolina quality traits in durum wheat breeding populations. Plant Genome.

[100] Sarup P., Jensen J., Ostersen T., Henryon M., Sørensen P. (2016). Increased prediction accuracy using a genomic feature model including prior information on quantitative trait locus regions in purebred Danish Duroc pigs. BMC Genet..

[101] Zuo J., Li J. (2014). Molecular genetic dissection of quantitative trait loci regulating rice grain size. Annu. Rev. Genet..

[102] Brinton J., Simmonds J., Minter F., Leverington-Waite M., Snape J., Uauy C. (2017). Increased pericarp cell length underlies a major quantitative trait locus for grain weight in hexaploid wheat. New Phytol..

[103] Guan P., Di N., Mu Q., Shen X., Wang Y., Wang X., Yu K., Song W., Chen Y., Xin M. (2019). Use of near-isogenic lines to precisely map and validate a major QTL for grain weight on chromosome 4AL in bread wheat (*Triticum aestivum* L.). Theor. Appl. Genet..

[104] Xing Y., Zhang Q. (2010). Genetic and molecular bases of rice yield. Annu. Rev. Plant Biol..

[105] Zhang W., Li H., Zhi L., Su Q., Liu J., Ren X., Meng D., Zhang N., Ji J., Zhang X. (2020). Functional markers developed from *TaGS3*, a negative regulator of grain weight and size, for marker-assisted selection in wheat. Crop J..

[106] Ramya P., Chaubal A., Kulkarni K., Gupta L., Kadoo N., Dhaliwal H.S., Chhuneja P., Lagu M., Gupta V. (2010). QTL mapping of 1000-kernel weight, kernel length, and kernel width in bread wheat (*Triticum aestivum* L.). J. Appl. Genet..

[107] Wang J., Liu W., Wang H., Li L., Wu J., Yang X., Li X., Gao A. (2011). QTL mapping of yield-related traits in the wheat germplasm 3228. Euphytica.

[108] Nezhad K.Z., Weber W.E., Röder M.S., Sharma S., Lohwasser U., Meyer R.C., Saal B., Börner A. (2012). QTL analysis for thousand-grain weight under terminal drought stress in bread wheat (*Triticum aestivum* L.). Euphytica.

[109] Zhang K., Wang J., Zhang L., Rong C., Zhao F., Peng T., Li H., Cheng D., Liu X., Qin H. (2013). Association analysis of genomic loci important for grain weight control in elite common wheat varieties cultivated with variable water and fertiliser supply. PLoS ONE.

[110] Sun C.W., Zhang F.Y., Yan X.F., Zhang X.F., Dong Z.D., Cui D.Q., Chen F. (2017). Genome-wide association study for 13 agronomic traits reveals distribution of superior alleles in bread wheat from the Yellow and Huai Valley of China. Plant Biotechnol. J..

[111] Jiang Q., Hou J., Hao C., Wang L., Ge H., Dong Y., Zhang X. (2011). The wheat (*T. aestivum*) sucrose synthase 2 gene (*TaSus2*) active in endosperm development is associated with yield traits. Funct. Integr. Genom..

[112] Zhang L., Zhao Y.L., Gao L.F., Zhao G.Y., Zhou R.H., Zhang B.S., Jia J.Z. (2012). *TaCKX6-D1*, the ortholog of rice *OsCKX2*, is associated with grain weight in hexaploid wheat. New Phytol..

[113] Ma L., Li T., Hao C., Wang Y., Chen X., Zhang X. (2016). *TaGS5-3A*, a grain size gene selected during wheat improvement for larger kernel and yield. Plant Biotechnol. J..

[114] Wang S., Yan X., Wang Y., Liu H., Cui D., Chen F. (2016). Haplotypes of the *TaGS5-A1* gene are associated with thousand-kernel weight in Chinese bread wheat. Front. Plant Sci..

[115] Hanif M., Gao F.M., Liu J.D., Wen W.E., Zhang Y.J., Rasheed A., Xia X.C., He Z.H., Cao S.H. (2016). *TaTGW6-A1*, an ortholog of rice *TGW6*, is associated with grain and yield in bread wheat. Mol. Breed..

[116] Chang J., Zhang J., Mao X., Li A., Jia J., Jing R. (2013). Polymorphism of *TaSAP1-A1* and its association with agronomic traits in wheat. Planta.

[117] Sestili F., Pagliarello R., Zega A., Saletti R., Pucci A., Botticella E., Masci S., Tundo S., Moscetti I., Foti S. (2019). Enhancing grain size in durum wheat using RNAi to knockdown *GW2* genes. Theor. Appl. Genet..

[118] Simmonds J., Scott P., Brinton J., Mestre T.C., Bush M., Del Blanco A., Dubcovsky J., Uauy C. (2016). A splice acceptor site mutation in *TaGW2-A1* increases thousand grain weight in tetraploid and hexaploid wheat through wider and longer grains. Theor. Appl. Genet..

[119] Golan G., Ayalon I., Perry A., Zimran G., Ade-Ajayi T., Mosquna A., Distelfeld A., Peleg Z. (2019). *GNI-A1* mediates trade-off between grain number and grain weight in tetraploid wheat. Theor. Appl. Genet..

[120] Sakuma S., Golan G., Guo Z., Ogawa T., Tagiri A., Sugimoto K., Bernhardt N., Brassac J., Mascher M., Hensel G. (2019). Unleashing floret fertility in wheat through the mutation of a homeobox gene. Proc. Natl. Acad. Sci. USA.

[121] Marcotuli I., Gadaleta A., Mangini G., Signorile A.M., Zacheo S.A., Blanco A., Simeone R., Colasuonno P. (2017). Development of a high-density SNP-based linkage map and detection of QTL for β-glucans, protein content, grain yield per spike and heading time in durum wheat. Int. J. Mol. Sci..

[122] Murai K., Miyamae M., Kato H., Takumi S., Ogihara Y. (2003). *WAP1*, a wheat *APETALA1* homolog, plays a central role in the phase transition from vegetative to reproductive growth. Plant Cell Physiol..

[123] Zhao X.Y., Liu M.S., Li J.R., Guan C.M., Zhang X.S. (2005). The wheat *TaGI1*, involved in photoperiodic flowering, encodesan Arabidopsis *GI* ortholog. Plant Mol. Biol..

[124] Guo J., Dai S., Li H., Liu A., Liu C., Cheng D., Cao X., Chu X., Zhai S., Liu J. (2018). Identification and expression analysis of wheat *TaGF14* Genes. Front. Genet..

[125] Feng N., Song G., Guan J., Chen K., Jia M., Huang D., Wu J., Zhang L., Kong X., Geng S. (2017). Transcriptome profiling of wheat inflorescence development from spikelet initiation to floral patterning identified stage-specific regulatory genes. Plant Physiol..

[126] Subira J., Álvaro F., Del Moral L.F.G., Royo C. (2015). Breeding effects on the cultivar× environment interaction of durum wheat yield. Eur. J. Agron..

[127] Qaseem M.F., Qureshi R., Shaheen H. (2019). Effects of pre-anthesis drought, heat and their combination on the growth, yield and physiology of diverse wheat (*Triticum aestivum* L.) genotypes varying in sensitivity to heat and drought stress. Sci. Rep..

[128] Fahad S., Bajwa A.A., Nazir U., Anjum S.A., Farooq A., Zohaib A., Sadia S., Nasim W., Adkins S., Saud S. (2017). Crop production under drought and heat stress: Plant responses and management options. Front. Plant Sci..

[129] Cammarano D., Ceccarelli S., Grando S., Romagosa I., Benbelkacem A., Akar T., Al-Yassin A., Pecchioni N., Francia E., Ronga D. (2019). The impact of climate change on barley yield in the Mediterranean basin. Eur. J. Agron..

[130] Gupta P.K., Balyan H.S., Gahlaut V., Kulwal P.L. (2012). Phenotyping, genetic dissection, and breeding for drought and heat tolerance in common wheat: Status and prospects. Plant Breed. Rev..

[131] El Hassouni K., Belkadi B., Filali-Maltouf A., Tidiane-Sall A., Al-Abdallat A., Nachit M., Bassi F.M. (2019). Loci controlling adaptation to heat stress occurring at the reproductive stage in durum wheat. Agronomy.

